# What is the Hidden Biological Mechanism Underlying the Possible SARS-CoV-2 Vertical Transmission? A Mini Review

**DOI:** 10.3389/fphys.2022.875806

**Published:** 2022-05-05

**Authors:** Rosa Sessa, Emanuela Anastasi, Gabriella Brandolino, Roberto Brunelli, Marisa Di Pietro, Simone Filardo, Luisa Masciullo, Gianluca Terrin, Maria Federica Viscardi, Maria Grazia Porpora

**Affiliations:** ^1^ Department of Public Health and Infectious Diseases, Microbiology Section, Sapienza University of Rome, Rome, Italy; ^2^ Department of Experimental Medicine, Sapienza University of Rome, Rome, Italy; ^3^ Department of Maternal and Child Health and Urology, Sapienza University of Rome, Rome, Italy

**Keywords:** SARS-CoV-2, coronavirus, placenta, pregnancy, vertical transmission, neonatal infection, pregnancy outcomes

## Abstract

Severe Acute Respiratory Syndrome Coronavirus 2 (SARS- CoV-2) represents an emerging infection that is spreading around the world. Among susceptible patients, pregnant women are more likely to develop serious complications and negative obstetric outcomes. Vertical transmission constitutes a debating issue which has not been completely understood. This review aims at describing the currently available evidence on SARS-CoV2 vertical transmission. We carried out a computerized literature search in the Cochrane Library, PubMed, Scopus and Web of Science, selecting the most relevant studies on vertical transmission from the outbreak onset until February 2022. The analysis of the available literature identifies the presence of SARS-CoV2 genome in different biological specimens, confirming the hypothesis that a transplacental infection can occur. In spite of the high number of infected people around the world, mother-to-child infections have been infrequently reported but it can be observed under certain biologic conditions. A deep knowledge of the underlying mechanisms of SARS-CoV2 vertical transmission is of paramount importance for planning an adequate management for the affected mothers and newborns.

## Introduction

Severe Acute Respiratory Syndrome Coronavirus 2 (SARS-CoV-2) was first identified in Wuhan region, China, on 31 December 2019, and the number of reported cases grew exponentially throughout the world. On 30 January 2020, the World Health Organization (WHO) declared a state of public emergency (World Health Organization, 2020)*.*

The most typical symptoms include fatigue, headache, cough, fever and diarrhea, which can be worsened by preexisting risk factors such as hypertension, diabetes, cardiovascular and respiratory diseases (Huang et al., 2020). Among the susceptible patients, pregnant women are more likely to have serious complications and an increased mortality rate (Hantoushzadeh et al., 2020; Villar et al., 2021). The physiological adaptations of the respiratory, cardiovascular, and immune systems during pregnancy may be responsible for a high risk of developing a severe illness and acute response to viral infections (Piccinni and Romagnani, 1996; Ramsey and Ramin, 2001; Druckmann and Druckmann, 2005; Hall and Klein, 2017)*.* Although CT scan represents the gold standard to evaluate the pulmonary involvement, in pregnant women lung ultrasound is considered a reliable method, minimizing the exposure to ionizing radiations. (Buonsenso et al., 2020; Fang et al., 2020; Gargani et al., 2020; Moro et al., 2020; Porpora et al., 2021).

Nowadays, a variety of therapeutic options exists, including antiviral anti-inflammatory and anti-oxidative drugs, monoclonal antibodies, and immunomodulatory agents (Filardo et al., 2020; Coopersmith et al., 2021; Cascella et al., 2022). To date, the most crucial step in containing SARS-CoV-2 pandemic is vaccination, which can markedly reduce adverse outcomes, hospitalization and deaths (Abu-Raya et al., 2021; Weinberger, 2021; Cascella et al., 2022).

The possibility of vertical transmission represents one of the most debated topics. Deep knowledge of this issue is of paramount importance for planning adequate management for the affected mothers and newborns.

To assess the biological mechanisms underlying the possible vertical transmission, we carried out a computerized literature search in the Cochrane Library, PubMed, Scopus and Web of Science databases, identifying the most relevant articles written in English from the onset of SARS-CoV-2 outbreak.

## Pregnancy Outcomes After SARS-CoV-2 Infection

Several studies have found a clear association between the infection of SARS-CoV-2 in pregnancy and the increasing risk of fetal malformations, preterm birth, intrauterine growth restriction, hypertensive disorders, and new onset gestational diabetes, as reported in Table 1 (Dashraath et al., 2020; Thomas et al., 2020; Timircan et al., 2021; Jamieson and Rasmussen, 2022), while a clear correlation between infection, stillbirths and miscarriages is still debated (Dashraath et al., 2020; Karimi-Zarchi et al., 2020; Khalil et al., 2020; Mendoza et al., 2020; Smith et al., 2020; Jamieson and Rasmussen, 2022). The pathophysiological mechanisms on pregnancy outcomes seem to involve different mediators.

**TABLE 1:** Pregnancy outcomes associated to SARS-CoV-2 infection and respective incidence.

Fetal malformations	Sahin et al. (2021)	0.5%
Preterm Birth	Delahoy et al. (2020)	12.6%
	Khoury et al. (2020)	18.8%
	Li M. et al. (2020)	16.7%
	London et al. (2020)	17.6%
	Wang M. J. et al. (2020)	16.9%
	Yang H. et al. (2020)	16%
	Jering et al. (2021)	7.19%
IUGR	Badhur et al. (2020)	11.7%
	Delahoy et al. (2020)	1.9%
Hypertensive disorders	Ahlberg et al. (2020)	7.74%
	Delahoy et al. (2020)	12%
	Wang W. et al. (2020)	18.8%
	Jering et al. (2021)	8.84%
Gestational diabetes	Delahoy et al. (2020)	11%
	Yan et al. (2020)	7.8%
	Patberg et al. (2021)	6.5%
Miscarriages	Yan et al. (2020)	12.5%
	Sahin et al. (2021)	2.2%
Stillbirths	Khoury et al. (2020)	0.8%
	Jering et al. (2021)	0.5%

Conde-Agudelo and Romero described the pathophysiological processes causing hypertensive complications and preeclampsia/eclampsia in patients with SARS-CoV-2. The viral binding process to the host cells led to a reduced activation of the renin angiotensin system: this phenomenon predisposes to the onset of preeclampsia, by decreasing the vasodilatory action of angiotensin 1 to 7 and increasing the vasoconstriction and inflammation.(Conde-Agudelo and Romero, 2022). Moreover, this proinflammatory environment is further enhanced by SARS-CoV-2 ability to shatter the syncytiotrophoblast, with a consequent increase of soluble fms-like tyrosine kinase-1 (sflt-1) which is strictly associated to the onset of this disease (Conde-Agudelo and Romero, 2022). Seethy et al. reported alterations in several placental proteins, such as MFGE8, PLAT, or PAR2, involved in trophoblastic invasion, proliferation and differentiation. These mechanisms define a direct correlation between a severe course of the disease and an increased risk of hypertensive complications (Seethy et al., 2021). Eberle et al. reported a possible association of SARS-CoV-2 and new-onset gestational diabetes: the viral entrance in the pancreatic beta cells through the angiotensin-converting enzyme 2 (ACE2) receptors, may cause insulin deficiency and an increased risk of keto-acydosis (Eberle et al., 2021). Furthermore, it seems that SARS-CoV-2 might cause pleiotropic alterations of glucose metabolism in patients with pre-existing diabetes or insulin-resistance (Rubino et al., 2020). Maternal infection seems to have a negative impact on neonatal health and an increased rate of preterm birth, stillbirth and low birth weight was reported, regardless of the mode of delivery (Kyle et al., 2022), probably due to the massive inflammatory response and to the suboptimal environment for the fetal growth (Wei et al., 2021).

Due to the negative effect of this infection on pregnancy, considerable support to pregnant patients comes from the implementation of the vaccination campaign. The American College of Obstetricians and Gynecologists (ACOG), the Society for Maternal-Fetal Medicine, the Centers for Disease Control and Prevention (CDC), and the Italian Society Of Gynecology and Obstetrics (SIGO) encourage SARS-CoV-2 vaccination in pregnancy (ACOG, 2018; CDC, 2021; Sigo Position Paper, 2021; European Center for Disease Prevention and Control, 2020), which also provides an immunological protection for the newborn (Jorgensen et al., 2022). In fact, pregnant women generate robust humoral immunity after the mRNA anti SARS-CoV2 vaccination (Gray et al., 2021; Shimabukuro et al., 2021). SARS-CoV-2 specific antibodies are present in both maternal and cord blood, suggesting that antibodies elicited by SARS-CoV-2 immunization cross the placenta (Beharier et al., 2021; Collier et al., 2021; Gray et al., 2021; Mithal et al., 2021). Both spike (S) and receptor binding domain (RBD) IgG antibodies as well as neutralizing antibodies are transplacentally transferred to the newborns, suggesting vaccines as the most important intervention to protect pregnant and breastfeeding women and their offspring from the infection (Jorgensen et al., 2022). To date, one of the most debating issues is to determine the most appropriate timing to assure an effective protection for this dyad. Recent data have, indeed, suggested that receiving a 2-dose mRNA COVID-19 vaccine during pregnancy can protect infants aged <6 months against the risk of COVID-19 hospitalization: this phenomenon seems to be enhanced among mothers vaccinated later in pregnancy (Dick et al., 2022). On this regard, a considerable decrease in anti-SARS-CoV-2 antibody levels has been observed throughout pregnancy in women vaccinated at early gestation, hinting at a relevant impact of the antenatal immunization timing on SARS-CoV-2 transplacental antibody transfer, which can potentially influence neonatal seroprotection (Rottenstreich et al., 2022).

## Placental Involvement in the Vertical Transmission of SARS-CoV2

Given the high number of cases of SARS-CoV-2 infection, it is essential to clarify the pathophysiological mechanisms that predispose to viral infection or protect the maternal-fetal interface. SARS-CoV-2 shares some structural characteristics with SARS and MERS, such as a positive single-stranded RNA virus with a nucleocapsid and an envelope and four structural proteins: spike (S), envelope (E), nucleocapsid (N) and membrane (M) (Guo et al., 2020; Villalain et al., 2020). N protein is essential to protect the RNA, whereas S, M, and E proteins compose the viral envelope (Kumar and Al Khodor, 2020).

The placental interface plays an essential role in protecting the fetus from different pathogens. The chorionic villi are composed of three kinds of trophoblasts: syncytiotrophoblast (STB), extravillous trophoblast (EVT), and cytotrophoblast (CTB). STB represents the outer layer and is in direct contact with maternal blood, mediating the exchange between mother and fetus; the CTB is the source of trophoblastic stem cells, and it is in close contact with STB. EVT is composed of cells at the base of stem villi and a disruption of its continuity can lead to the placental passage of pathogens and fetal infection (Maltepe and Fisher, 2015; Pereira, 2018).

**FIGURE 1:**
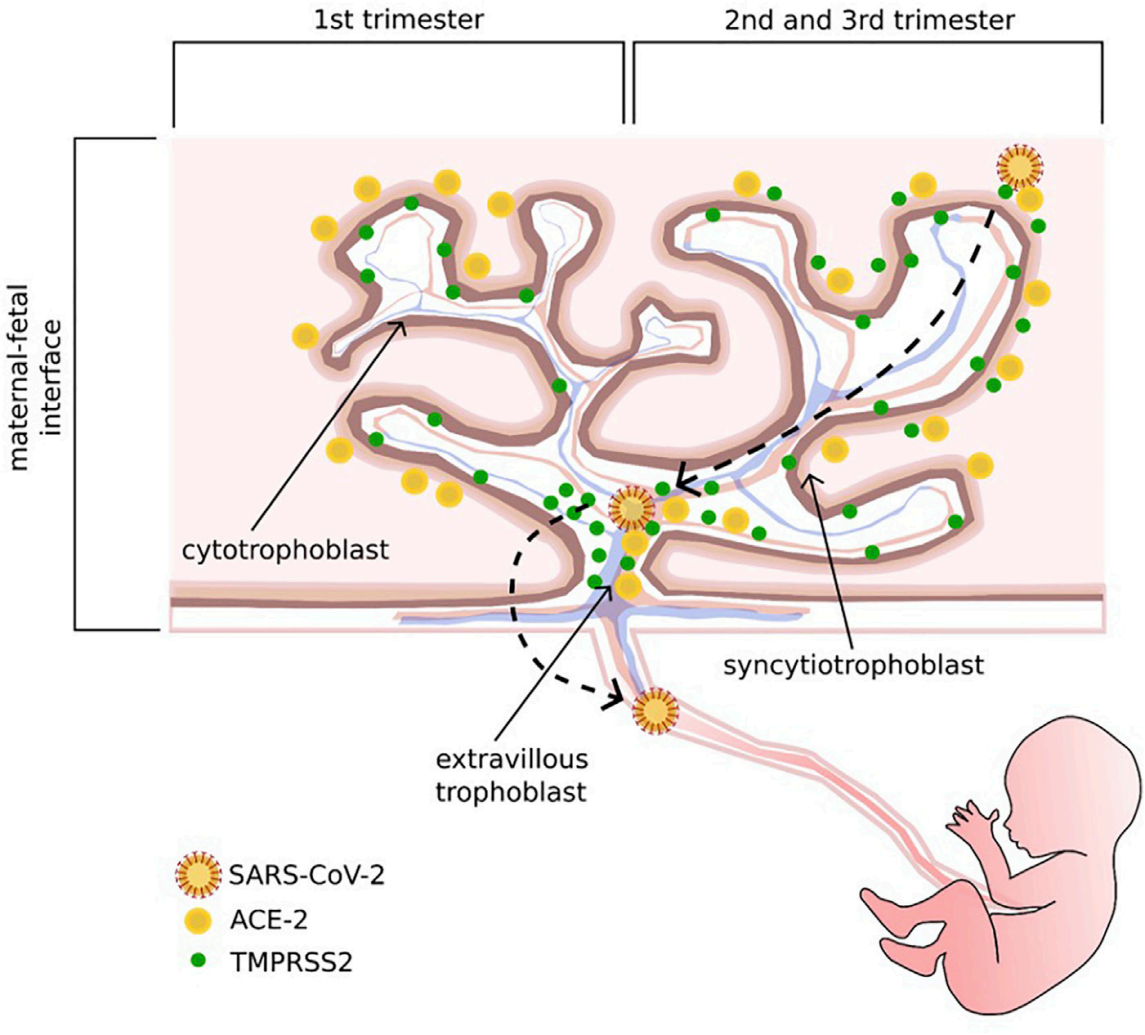
Schematic representation of ACE 2 and its co-receptor, TMPRSS 2, distribution through trimesters and trophoblast layers, and the potential adhesion and invasion pathways of SARS-CoV-2. In the first trimester, ACE2 is preferentially expressed in the syncytiotrophoblast layer, while TMPRSS2 is mostly localized on the cytotrophoblast and extravillous trophoblast layers; in the second and third trimesters, ACE2 is also expressed on the extravillous trophoblast layer, and TMPRSS2 can be found on all trophoblast sites.

Spike glycoprotein is essential to promote the attachment of SARS-CoV-2 to the surface of the host cell and the subsequent fusion of the viral envelope. The target receptor is ACE2 which needs the serine proteases TMPRSS2 as a co-receptor to finalize the cell invasion (Valdés et al., 2006; Kumar and Al Khodor, 2020), considered as one of the most important factors contributing to its pathogenicity (Hoffmann et al., 2020; Zhang et al., 2020). ACE2 receptor is a transmembrane peptidase expressed on the surface of lungs, kidneys, intestinal tract, and placental tissues (Hoffmann et al., 2020). The placental immunohistochemical analysis revealed a different ACE2 and TMPRSS2 distribution through trimesters and trophoblast layers. During the first trimester, ACE2 is preferentially expressed in STB and TMPRSS2 in EVT and CTB. In the second and third trimesters, ACE2 expression also involves EVT, while TMPRSS2 is equally represented in all trophoblast sites (Cui et al., 2021). (Figure 1). The pleiotropic expression of ACE2 and TMPRSS2 hints at the biological basis of SARS-CoV-2 transplacental route. Further evidence reported the presence of SARS-CoV-2 S- and N- protein expression in the STB layer, confirmed by in-situ hybridization techniques, showing the presence of viral RNA (Baud et al., 2020). The low prevalence of SARS-CoV-2 RNA in placental tissue found in some studies could be explained by the focal localization of the virus in this organ, which may influence the detection of viral RNA. This problem could be overcome by testing more samples from the same placenta. A co-expression of ACE2 and TMPRSS2 was found in stromal and perivascular decidual cells and in villous CTB and STB (Li M. et al., 2020). Several histologic alterations of placental tissue, such as massive vascular malperfusion, chronic intervillositis, funisitis and villitis seem to be a potential sign of subsequent fetal infection (Schwartz et al., 2020). Shanes et al. reported a high rate of placental vascular malperfusion in 16 placentas of women with COVID-19 who delivered in the third trimester as compared to unaffected patients, despite the negative results of RT-PCR for SARS-CoV-2 (Shanes et al., 2020).

The histopathologic alterations of the placenta and the presence of viral proteins strongly support the possibility of the transplacental passage of the virus: in fact, placental damage may be considered as an indirect sign of a maternal-fetal response to viral infection (Baud et al., 2020; Hosier et al., 2020).

In support to the link between histopathological damage and SARS-CoV-2 infection of placental tissue, it stands the identification of a strong expression of SARS-CoV-2 S- and N-proteins in the STB layer, with an increased expression of S- protein where the intervillous inflammation was huge (Facchetti et al., 2020). This was confirmed by *in-situ* hybridization, detecting the presence of viral RNA in the syncytiotrophoblast. At the same time, the placenta showed signs of intervillositis, necrosis of the syncytiotrophoblast, evidence of fetal vascular malperfusion and fibrin deposition (Facchetti et al., 2020).

The cellular apoptosis and the vascular impairment may enhance the placental permeability (Miranda et al., 2019), predisposing to circulatory disturbances that lead to subchorionic and intervillous fibrin deposition (Baergen and Heller, 2020; Shanes et al., 2020).

## Evidence of Vertical Transmission and Neonatal Infection

The risk of vertical transmission was analyzed during previous coronavirus outbreaks without conclusive results (Hospital et al., 2003; Wong et al., 2004; Akhtar et al., 2020)*.*

Analyzing different biological specimens and the presence of antibodies in the maternal blood, several studies pointed to the transplacental passage of SARS-CoV-2 viral particles through the maternal-fetal interface, with an estimated rate of vertical transmission ranging from 3 to 8% of cases (Bwire et al., 2021; Kotlyar et al., 2021; Taglauer et al., 2022). Concerns over SARS-CoV-2 vertical transmission are appropriate, due to the tropism for the cells of the maternal-fetal interface (Li N. et al., 2020). It is well-known that a viral infection, acquired during pregnancy, can lead to three different vertical transmissions: intrauterine, intrapartum and postpartum routes (Schwartz, 2020). The transmission *in utero* occurs when the virus, circulating in the bloodstream, crosses the maternal-placental interface, reaching the umbilical cord and infecting the fetus (Mahyuddin et al., 2020). Intrapartum infection may occur during labor when the fetus is exposed to a pathogen that has colonized vaginal secretions and/or feces (Schwartz et al., 2020). Postpartum transmission happens after delivery, when the infection can spread through fomites and maternal close contact. A retrospective study conducted by Ferrazzi et al., noticed that infected women who breastfed without wearing a mask can facilitate neonatal infection (Ferrazzi et al., 2020). Since the available evidence does not support breast milk as a source of neonatal infection (Marín Gabriel et al., 2020), it seems that, whether hygienic measures are not assured, the close connection between mother and infant after birth might represent a viable way for the transmission of pathogen’s particles (Marín Gabriel, M. Á. et al., 2020, Stiehm and Keller, 2001). In this way the virus can reach the target cells invading the infant’s gastrointestinal and respiratory system. Regarding breastfeeding as a carrier for the infection, a recent review reported no replication-competent SARS-CoV-2 in breastmilk (Jamieson and Rasmussen, 2022).

To assess the most viable route of fetal transmission, Beesley et al. conducted a retrospective study evaluating the gene expression level of ACE2 and TMPRSS2 through PCR analysis of multiple fetal tissues at different gestational ages during the second trimester. According to their results, the assumption for fetal infection is the co-expression of both target proteins, which seems to occur only in the fetal kidney and gastrointestinal tract. Therefore, it is possible to hypothesize a viral entry into the bowel lumen through fetal swallowing of infected amniotic fluid (Beesley et al., 2022).

Alternative mechanisms of cellular entry were also proposed. In particular, an antibody-dependent enhancement (ADE) of the virus infection was suggested. According to this theory, Fc receptors may facilitate cellular entry by inducing a conformational change in S protein, which promotes a proteolytic cleavage. The cleavage, in turn, allows the viral binding and the subsequent activation of dipeptidyl dipeptidase 4 (DDP4) receptors, which fosters cellular entry (Moore and Suthar, 2021).

SARS-CoV-2 virions in the syncytiotrophoblast and in the fetal capillary endothelium have been recently reported, supporting the hypothesis of the pathogen transfer through the entire thickness of the maternal-fetal interface (Di Gioia et al., 2022); however, neonatal infection rarely occurs. The low rate of vertical transmission is not always related to a reduction in ACE2 and TMPRSS2 expression but it is also due to changes occurring after the virus entry (Ouyang et al., 2021). Furthermore, experiments on cells’ cultures exposed to trophoblastic cell’s microenvironment showed a selective antiviral activity against different viruses like togavirus, varicella zoster and HIV. This action seems to be ineffective on other pathogens like *L. monocytogenes* and Toxoplasma gondii (Bayer et al., 2015). Human trophoblasts are resistant to different viruses by the intervention of a huge variety of cells, including miRNAs, located on chromosome 19, which are exclusively expressed in the placenta (Bortolin-Cavaille et al., 2009). Their targets include different types of non-trophoblastic cells like fibroblasts, Hofbauer cells, maternal and fetal cells. These miRNAs send silencing signals to nearby and distant cells through a non-hormonal mechanism (Schwartz, 2017). These findings support the hypothesis that, in some conditions, the trophoblastic cells and the miRNAs are able to inhibit the viral entrance in the cell and the subsequent perinatal infection. The largest published study, prospectively included 427 pregnant women with confirmed SARS-CoV-2 infection and 244 newborns. Among the babies, 12 had confirmed SARS-CoV-2 infection, but only six of them within 12 h after birth. The mode of delivery did not influence the viral transmission, as three infants born by elective cesarean section had evidence of both viral RNA in nasopharyngeal swabs and positive IgM circulating antibodies (Knight et al., 2020). The observation that several neonates born to positive mothers have IgM antibodies confirms the fetal viral infection, as IgM antibodies do not cross the placenta (Kotlyar et al., 2021). However, an early postnatal infection cannot be excluded.

By contrast, Yan et al., did not report any vertical transmission, confirmed by the absence of SARS-CoV-2 RNA in amniotic fluid, cord blood and pharyngeal swabs of all the newborns of 116 affected women (Yan et al., 2020). In addition, in a study on 38 pregnant women with SARS-CoV-2, no cases of intrauterine transmission were found, despite the presence of several neonatal complications, such as severe neonatal respiratory distress syndrome, pneumothorax, asphyxia, stillbirth or small fetus for gestational age (Schwartz, 2020). An overview of the evidence concerning SARS-CoV2 vertical transmission regardless of the mode of delivery is shown in Table 2.

**TABLE 2:** Studies on the vertical transmission of Sars-CoV-2. CR, case report; OS, observational study; PCS, prospective cohort study; RS, retrospective study; CaS, case series; VD, vaginal delivery; CS, cesarean section.

Author year	Type of study	n. patients	n. newborns	Gestational age (weeks + days)	Mode of delivery (n.)	Neonatal swab n.	Placenta n.	Cord blood n.	Amniotic fluid n.	Neonatal sierology n.
Alzamora et al. (2020)	CR	1	1	33	CS (1)	1/1	-	-	-	IgG 0/1 IgM 0/1
Baud et al. (2020)	CR	1	1	19	VD (1)	0/1	1/1	-	0/1	-
Blauvelt et al. (2020)	CR	1	1	28	CS (1)	0/1	-	-	-	IgG 0/1 igM 0/1
Buonsenso et al. (2020)	OS	7	2	8-37+3	CS (2)	0/2	1/2	1/2	0/7	IgG 1/1 IgM 0/1
Chen et al. (2020)	RS	3	3	35– 38+6	CS (3)	0/3	-	-	-	-
Dong et al. (2020)	CR	1	1	34+2	CS (1)	0/1	-	-	-	IgG 1/1 IgM 1/1
Ferrazzi et al. (2020)	RS	42	42	<37 - >37	VD (24) CS (18)	3/42	-	-	-	-
Gidolf et al. (2020)	CR	1	2	36	CS (1)	0/2	-	-	-	-
Govind et al. (2020)	CaS	9	9	27-39	VD (1) CS (8)	1/9	0/9	-	0/9	-
Hu et al. (2020)	CaS	7	7	37-40	VD (1) CS (6)	1/7	-	-	0/7	-
Kalafat et al. (2020)	CR	1	1	35+3	CS (1)	0/1	0/1	0/1	-	-
Khan et al. (2020)	CaS	17	17	35-41	CS (17)	2/17	-	-	-	-
Kirtsman et al. (2020)	CR	1	1	35	CS (1)	1/1	1/1	0/1	-	-
Knight et al. (2020)	PCS	427	244	<22 – 37+6	VD (101) CS (144)	12/244	-	-	-	-
Lang and Zhao (2020)	CR	1	1	35+2	CS (1)	0/1	0/1	0/1	0/1	-
Lee et al. (2020)	CR	1	1	36+2	CS (1)	0/1	0/1	0/1	0/1	-
Liu et al. (2020)	CaS	3	3	37-40	VD (1) CS (2)	0/3	-	0/3	-	-
Lowe and Bopp (2020)	CR	1	1	40+3	VD (1)	0/1	-	-	-	-
Lyra et al. (2020)	CR	1	1	39+4	CS (1)	0/1	-	-	-	-
Patané et al. (2020)	CaS	22	22	35-37	VD (11) CS (11)	2/22	-	-	-	-
Penfield et al. (2020)	CaS	31	11	26+4 – 41+2	VD (7) CS (4)	0/10	1/1	-	-	-
Pierce-Williams et al. (2020)	PCS	64	33	16+1 – 39+1	VD (8) CS (24)	1/33	-	-	-	
Quiancheng et al. (2020)	RS	28	23	36+5 – 39	VD (5) CS (17)	0/23	-	-	-	-
Schnettler et al. (2020)	CR	1	1	30+3	CS (1)	0/1	-	-	0/1	-
Vivanti et al. (2020)	CR	1	1	35+5	CS (1)	1/1	1/1	1/1	1/1	1/1
Wang W. et al. (2020)	CR	1	1	40	CS (1)	1/1	0/1	0/1	-	-
Xiong et al. (2020)	CR	1	1	33	VD (1)	0/1	-	-	0/1	0/1
Yan. et al. (2020)	RS	116	100	37+3 – 39+4	VD (14) CS (85)	-	0/10	0/10	-	-
Yang R et al. (2020)	RS	27	24	30-40	VD (5) CS (18)	0/23	-	-	-	IgG 1/1 IgM 1/1
Yu et al. (2020)	RS	7	7	37-41+5	CS (7)	1/3	-	-	-	-
Zamaniyan et al. (2020)	CR	1	1	32	CS (1)	0/1	0/1	0/1	1/1	-
Zeng et al. (2020)	CaS	6	6	-	CS (6)	0/6	-	-	-	IgG 3/6 IgM 2/6
Angelidou et al. (2021)	PCS	250	255	37-39	VD (142) CS (113)	5/255	-	-	-	-
Fan et al. (2021)	CaS	2	2	36-37	CS (2)	0/2	0/2	0/2	0/2	-
Peng et al. (2021)	CR	1	1	34+3	CS (1)	0/1	0/1	0/1	0/1	-
Celik et al. (2022)	PCS	30	31	20-36+1	VD (5) CS (25)	0/31	0/31	0/31	0/31	IgG – IgM 20/30
i Gioia et al. 2022	CR	1	1	36+1	VD (1)	0/1	1/1	-	-	-
Morales et al. (2022)	CR	1	2	32	CS (1)	1/2	1/2	-	-	0/2

## Discussion

Most available information on SARS-CoV-2 infection comes from adults and the effect of the infection on newborns is still controversial. During pregnancy, several complications were reported with a high rate of preterm birth, fetal malformations, intrauterine growth restriction, hypertensive disorders, and new onset gestational diabetes (Dashraath et al., 2020; Thomas et al., 2020; Timircan et al., 2021; Jamieson and Rasmussen, 2022). These outcomes are not always related to the severity of the maternal disease, since they may occur in the absence of symptoms. It was shown that ACE2 and TMPRSS2 receptors, the target sites for SARS-CoV-2 invasion, are expressed in the placenta (Jamieson and Rasmussen, 2022). Their expression varies in the different trimesters of pregnancy. Placental infection is responsible for circulatory abnormalities, which may provoke thrombosis of vessels and ischemic lesions, resulting in a chronic fetal hypoxia. In addition, the vascular damage and the inflammatory environment seem to be involved in preterm birth and preeclampsia. Despite the huge knowledge on maternal complications, there is fragmentary data on fetal and neonatal infection and on the biological mechanisms involved. Vertical transmission was observed in some cases but the majority of studies did not find the virus in the newborn. In some cases, the virus was detected in nasopharyngeal swabs, in others, circulating IgM were found, even in the presence of negative swabs, showing that the fetus was infected (Kotlyar et al., 2020). The absence of viral RNA in nasopharyngeal swabs raises the question whether it is appropriate to limit SARS-CoV-2 testing in infants to routine respiratory samples or, instead, it is necessary to examine samples from different sites. In fact, fetal infection could not be easily detected by pharyngeal swabs, since the virus mostly dwells in the gastrointestinal tract. Therefore, rectal swabs analysis at birth should always be performed. In addition, Liguoro et al. argue that newborns are often tested for SARS-CoV-2 only if symptomatic (Liguoro et al., 2021); this might reduce the possibility of detecting a vertical transmission. Indeed, the evidence on the tissue tropism of SARS-CoV-2 suggests the potential infection of other organs and tissues rather than the respiratory tract in infants, although the clinical outcomes are still unconfirmed.

The absence of a neonatal infection could be also due to the neutralizing effect of maternal immunity against the virus: in fact, studies on influenza confirmed the efficacy of the maternal humoral response in reducing the infection in infants (Puck et al., 1980; Zaman et al., 2008). The immune response could be related to the severity of disease; Szymczak reported that the production of anti-N antibodies is strongly associated with maternal severe symptoms and a rise in body temperature. Therefore, the more aggressive the disease is, the more effective the antibody response is (Szymczak et al., 2021). Moreover, it is often impossible to determine the exact time of the maternal infection, making it challenging to obtain data on SARS-CoV-2 prevalence in newborns.

There are several limitations in our review principally due to the quality of the available literature, as they are mainly case reports, case series or low-sample populations studies. Moreover, in most cases there is a lack of data on placenta, amniotic fluid and cord blood samples analysis. Lastly, the analysis of specific IgG and IgM in newborns’ blood, which are of paramount importance, are rarely reported.

To date, this narrative review includes the most recent and thorough evidence regarding the mechanisms underlying the possible SARS-CoV-2 vertical transmission. The long-term implications of the virus infections remain to be investigated. A better knowledge of the trophoblastic immune response and of the virus cell entry pathways is necessary to fully understand the mechanisms involved in susceptibility to SARS-CoV-2 infection. More research studies on larger cohorts of pregnant women, as well as longitudinal studies on their newborns, will be necessary to understand whether SARS-CoV-2 mother-to-child transmission could cause long-term consequences on maternal and fetal health.
